# Abnormally localized DLK1 interacts with NCOR1 in non-small cell lung cancer cell nuclear

**DOI:** 10.1042/BSR20192362

**Published:** 2019-12-13

**Authors:** Jinjing Tan, Susu Zhang, Lin Li, Jing Mu, Ziyu Wang, Lina Zhang, Mei Jiang, Weiying Li, Xin Yang, Yu Liu, Yanning Gao

**Affiliations:** 1 Department of Cellular and Molecular Biology, Beijing Chest Hospital, Capital Medical University and Beijing Tuberculosis and Thoracic Tumor Research Institute, Beijing 101149, China; 2 Department of Cancer Center, Beijing Chest Hospital, Capital Medical University and Beijing Tuberculosis and Thoracic Tumor Research Institute, Beijing 101149, China; 3 Department of Biochemistry, Vanderbilt University School of Medicine, Nashville, TN, U.S.A.; 4 State Key Laboratory of Molecular Oncology, Beijing Key Laboratory for Carcinogenesis and Cancer Prevention, National Cancer Center/Cancer Hospital, Chinese Academy of Medical Sciences and Peking Union Medical College, Beijing 100021, China; 5 Department of Pathology, National Cancer Center/Cancer Hospital, Peking Union Medical College and Chinese Academy of Medical Sciences, Beijing 100021, China; 6 Department of Pathology, Beijing Chest Hospital, Capital Medical University and Beijing Tuberculosis and Thoracic Tumor Research Institute, Beijing 101149, China; 7 Key Laboratory of Carcinogenesis and Translational Research (Ministry of Education), Department of Pathology, Peking University Cancer Hospital and Institute, Beijing 100142, China; 8 Pediatric Translational Medicine Institute, Shanghai Children’s Medical Center, Shanghai Jiao Tong University School of Medicine, Shanghai 200025, China

**Keywords:** DLK1, NCOR1, non-small-cell lung cancer, notch signalling pathway, nuclear localization, protein-protein interactions

## Abstract

Delta-like homolog 1 (DLK1) regulates noncanonical Notch signaling pathway as ligand. DLK1 was abnormally expressed in a variety of tumors, affecting tumorigenesis and developments. The biological function of DLK1 toward cell proliferation and signaling activation was controversial across different cell types. Two currently known isoforms of DLK1, which are membrane-tethered isoform and soluble isoform, are believed to be the key of DLK1 dual behaviors. While these isoforms are not enough to explain the phenomena, our observations offer the possibility of a third isoform of DLK1. In the present study, we verified the nuclear localization of DLK1 in lung cancer cells. The nuclear localized DLK1 was observed in 107 of 351 non-small cell lung cancer (NSCLC) samples and was associated with tissue differentiation and tumor size. Through co-immunoprecipitation (co-IP) combined mass spectrometry (MS), we identified nuclear receptor corepressor 1 (NCOR1) as DLK1’s novel interaction protein and confirmed their interaction in nuclear. We analyzed the expression of NCOR1 in two independent cohorts and demonstrated that NCOR1 is a tumor suppressor and has prognosis potential in lung squamous carcinomas. At last, we analyzed the colocalization of DLK1 and NCOR1 in 147 NSCLC samples by immunohistochemistry (IHC). The result indicated NCOR1 might participate with nuclear localized DLK1 in regulating cell differentiation.

## Introduction

Lung cancer is the cancer with the highest incidence and mortality rates for both genders worldwide [1,2]. Our precious studies based on mRNA micro-array analysis revealed that Delta-like homolog 1 (DLK1) was up-regulated expressed in human lung squamous cell carcinoma (SCC/LUSC) [3].

DLK1, in short for the Delta-like 1 homolog, also named as the pre-adipocyte factor 1 (Pref-1) [4] or fetal antigen-1 (FA1) [5], is an essential and widely expressed protein during human embryonic period. Yet, DLK1 expression decreases along with increased cell differentiation as gestation proceeds and in most tissues DLK1 is absent around birth. In adults, expression of DLK1 is restricted to a few tissues and progenitor cells, but is re-expressed during disease and regeneration [6]. In our previous studies, we found that up-regulated DLK1 expression is associated with lung cancer cell invasion through activating Notch signaling [7]. DLK1 was also found aberrantly expressed in other human cancers, including hepatocellular carcinoma (HCC) [8], breast cancer (BC) [9], acute myeloid leukemia (AML) [10] and so on. According to these studies, DLK1 could promote tumorigenesis and invasion [7,11]. Yet, the vast majority of studies other than cancer fields, however, reported DLK1 to inhibit cell proliferation [12–15].

DLK1 is a transmembrane protein, whose extracellular domain can be cleaved and released as a soluble isoform. For this reason, researches of DLK1 mainly focus on its membrane-tethered and soluble isoforms. Nevertheless, we observed positive staining of DLK1 in cancer cell nuclear during immunohistochemistry (IHC) experiments on non-small cell lung cancer (NSCLC) tissues. If we look closely at the images in earlier published articles, it was worth noting that Wilms tumors and HCC cell lines also obtained DLK1 nuclear staining in IHC assay [16,17]. Unfortunately, researchers chose to ignore this phenomenon only because DLK1 has no nuclear localization signal (NLS) and the nuclear localization of DLK1 does not show biological meaning in their data. The nuclear localization of DLK1 in our data revealed great clinical significance for lung cancer. Therefore, we needed to prove the reality of our observation and explore the mechanism behind.

In the current study, we examined the localization of DLK1 in both NSCLC tissues and cell lines and analyzed the clinical feature of its expression. To explore the function of nuclear localized DLK1, we started by screening DLK1 interaction partners, from which we identified and characterized a novel interacting protein of DLK1, NCOR1 (nuclear receptor corepressor 1). The clinical roles of NCOR1 was assessed in NSCLC from both local data and public databases. The down-regulation of NCOR1 was associated with prognosis in patients with lung cancer. Furthermore, we analyzed the clinical significance of different colocalization conditions of DLK1 and NCOR1 in nucleus. At last, our study has confirmed the nuclear localization of DLK1 in NSCLC and identified a novel interacting protein of DLK1, which might be involved in regulating cell differentiation.

## Materials and methods

### Patients, specimens and cell lines

Two hundred and four paired NSCLC FFPE tissues were collected from the Cancer Hospital, Peking Union Medical College (PUMC) and Chinese Academy of Medical Science (CAMS). Tumor tissue microarrays (TMAs) consisting of 150 paired NSCLC tissues and adjacent normal tissues were purchased from Shanghai OUTDO Biotech (Shanghai OUTDO Biotech Co., Shanghai, China). All sample donors provided informed consent and the study was conducted in accordance with the China Ethical Review Committee. NSCLC cell lines (A549 and H1299) were obtained from the American Type Culture Collection (ATCC; Manassas, VA, U.S.A.). Cells were cultured in RPMI-1640 medium (Gibco, Grand Island, NY) supplemented with 10% fetal bovine serum (FBS; Hyclone, Logan, UT, U.S.A.) in a humidified atmosphere with 5% CO_2_ at 37°C.

### IHC analysis

IHC staining of DLK1 was performed on both FFPE tissue sections and TMAs. IHC staining of NCOR1 was performed on TMAs matched with DLK1. Briefly, the antibodies were diluted at appropriate concentration and incubated with the sections at 4°C overnight. Biotinylated goat anti-rabbit IgG secondary antibody was applied for 30 min, followed by incubation with the avidin–biotin and streptavidin complex. Nuclei were counterstained blue with Hematoxylin. The slides were scored by two experienced pathologists, independently. Since the DLK1 was generally considered to be located in cytoplasm or membrane, the expression of DLK1 was scored only based on the intracytoplasmic staining.

### Antibodies

For all the immunological hybridization experiments, the following primary antibodies were used: rabbit polyclonal anti-DLK1 (P10636; Proteintech Group), anti-NCOR1 (ab24552; Abcam), normal rabbit IgG (#2729; Cell Signaling Technology), mouse mono anti-NCOR1 (NBP1-28863; Novus Biologicals). A monoclonal mouse anti-β-actin (sc-7210; Santa Cruz Biotechnology) or anti-GAPDH (sc-137179; Santa Cruz Biotechnology) antibody was used as a marker of equal loading. Goat polyclonal anti-mouse IgG-horseradish peroxidase (HRP) (ab205719; Abcam), anti-mouse IgG-Alexa Fluor® 555 (#4409; Cell Signaling Technology), anti-rabbit IgG-HRP (ab6721; Abcam) anti-rabbit IgG-Alexa Fluor® 488 (#4412; Cell Signaling Technology) were used as secondary antibodies. All of the primary and secondary antibodies used in the present study were diluted in a PBS-0.1% Tween 20 solution containing 5% skim milk or 3% BSA.

### Transfection and plasmid

A549 cell line was transiently transfected with an empty vector (A549-vector) or with pcDNA3.1 plasmid that contains the *DLK1* gene (A549-dlk1), which was constructed previously [7]. Briefly, cells (5 × 10^5^) were seeded on six-well plates in complete medium; waited until the cells were attached to the bottom, which was usually the following day, transfected the cells with the liposome transfection reagent (Lipofectamine™ 3000; Thermo Fisher Scientific, U.S.A.). The plasmid DNA and the liposome were diluted into two solutions of medium without serum; the mixture was incubated for 20 min and then added to cells.

### Nuclear protein extraction and Western blot

Nuclear protein and cytoplasmic protein was extracted separately using NE-PER™ Nuclear and Cytoplasmic Extraction Reagents (Thermo Scientific, U.S.A.) according to the product manual. Western blot was performed using the standard techniques. In brief, cells were lysed in RIPA contacting PMSF and protease inhibitor cocktail; cell lysates were denatured loading buffer for 5 min in 100°C and run on 10% SDS/PAGE gel. The proteins in gel then were transferred to PVDF membranes. The membranes were blocked by 5% skim milk PBS solution, probed with specific antibodies, and developed using ECL blotting substrate. β-actin or GAPDH was used as loading control.

### Immunofluorescence and confocal imaging

The cells were seeded on coverglass placed in six-well plates. Cells then were fixed in cold methanol at 4°C for 10 min and permeabilized in 0.5% Triton X-100 PBS solution 4°C for 10 min. After several washings by PBST, cells were blocked in 5% skim milk PBST 1 h at room temperature. For single label, cells were incubated with rabbit polyclonal anti-DLK1 antibody at 4°C overnight. For double label, cells were incubated with rabbit polyclonal anti-DLK1 antibody and mouse monoclonal anti-NCOR1 antibody together at 4°C overnight. They then were washed three times in PBST and incubated with goat anti-rabbit Alexa Fluor® 488–conjugated antibody (DLK1) for 1 h and/or with goat anti-mouse Alexa Fluor® 555–conjugated antibody (NCOR1) for 1 h. Finally, after washing in PBST, cells were incubated with DAPI to visualize the nuclei. The coverglass were mounted face down on an air-dried glass slide using antifading mounting medium (Solarbio, Beijing). The slides were viewed on Zeiss Laser-scanning confocal microscope equipped with Zeiss image processing software (TCL SP8).

### DLK1 pull-down assay and mass spectrometry analysis

Cell lysates were incubated with DLK1 antibody and protein A/G beads (Thermo Fisher, U.S.A.) overnight at 4°C. Then beads were collected by centrifugation and washed by PBS. DLK1-associated proteins were eluted and resolved by SDS/PAGE followed by Coomassie Brilliant Blue staining (Bio-Rad, U.S.A.). To identify specific DLK1 interactors, paired differential bands of anti-DLK1 and isotype antibody control eluates were selected and cut for LC-MS analysis. Two independent pull-down experiments were performed. The proteins were sequenced with at least two peptides and scores more than 22 were considered as reliable identification. Using normal rabbit IgG as negative control, non-specific binding proteins were excluded.

### Co-immunoprecipitation and Western blot

Cells were washed in PBS, collected by centrifugation and lysed in RIPA contacting PMSF and protease inhibitor cocktail. Cell lysate was collected by centrifugation, pre-cleared by incubation with protein A/G beads (Thermo Fisher, U.S.A.), incubated with the primary antibody and further incubated with protein A/G beads on a rotational platform, centrifuged and the supernatant and the beads were collected separately. Beads were washed and resuspended in SDS loading buffer. Immunoprecipitated proteins were separated by 10% SDS/PAGE gel. Western blot was performed as described above.

### Bioinformatics analysis

RNA-sequencing data of human LUSC and lung adenocarcinoma (ADC/LUAD) was downloaded from The Cancer Genome Atlas (TCGA). The TCGA dataset containing 1016 tumor samples and 127 normal samples, which included FPKM value of mRNA expression data and corresponding clinical information. Survival analysis was performed using Kaplan–Meier method. Wilcox.test was used to analyze the statistic difference in NCOR1 expression between groups. All analysis and drawing were done by R software (3.5.2).

## Results

### Overexpression of DLK1 in NSCLC

**Figure 1:**
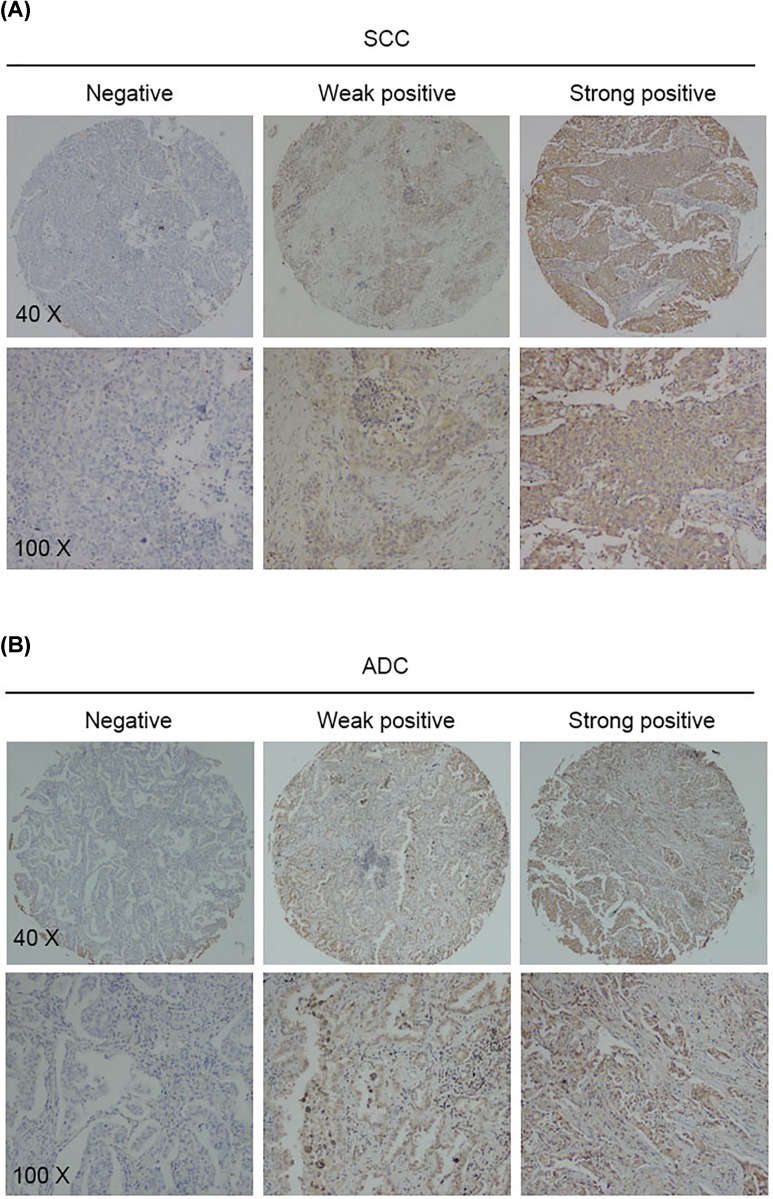
DLK1 is overexpressed in NSCLC tissues Representative staining of DLK1 IHC in lung SCC (**A**) and ADC (**B**) tissue microarrays (TMA).

IHC of DLK1 was performed on 351 NSCLC specimens, including 174 SCC tissues and 177 ADC tissues. DLK1 was found to be significantly up-regulated in 126 (72.4%) SCC specimens and 137 (77.4%) ADC specimens, whereas the expression was hardly detected in adjacent non-cancerous bronchial epithelium (48/156; 30.8%) or alveolus (7/181; 3.9%) (Figure 1 and Table 1). Among the 126 DLK1-positive SCC tissues, 77 (44.3%) SCC specimens showed weak DLK1 expression, 49 (28.2%) displayed strong reactions. While in ADC tissues, 97 (54.8%) and 40 (22.6%) specimens showed weak and high DLK1 expression, respectively, which was in consensus with the tendency in SCC. We investigated the correlation of DLK1 expression and the clinical feature of the patients, including patients’ age, gender, tumor stage, tumor size, node metastasis status and cell differentiation degree. In SCC, the overexpression of DLK1 was correlated with tumor stage (*P*=0.008), node metastasis (*P*=0.007) and differentiation (*P*=0.007) (Table 2). Tumors with advanced stage or positive node metastasis status or poor cell differentiation would have higher DLK1 expression. However, such pattern was not found in ADC samples (Table 3).

**Table 1:** The expression of DLK1 in NSCLC specimens

Histological type	Immunohistochemical scoring of DLK1, *n* (%)			Total	*P*^1^
	Negative	Weak	Strong		
Bronchial epithelia	108 (69.2)	23 (14.7)	25 (16.0)	156	
Primary lung SCC	48 (27.6)	77 (44.3)	49 (28.2)	174	<0.001^2^
Alveolus	174 (96.1)	7 (3.9)	0 (0.0)	181	
Primary lung ADC	40 (22.6)	97 (54.8)	40 (22.6)	177	<0.001^3^

1 The expression of DLK1 in primary tumors versus normal lung tissues was significant, *P*<0.05 with χ^2^ test. Notably, the morphologically normal tissues were collected from the same group of lung cancer patients.

2 Primary lung SCC vs normal bronchial epithelia.

3 Primary lung ADC vs normal alveolus.

**Table 2:** The expression of DLK1 versus clinical features in SCC

Clinicopathology	Immunohistochemical scoring of DLK1, *n* (%)			Total	*P*^1^
	Negative	Weak	Strong		
Gender					
Male	45 (27.6)	73 (44.8)	45 (27.6)	163	0.800
Female	3 (27.3)	4 (36.4)	4 (36.4)	11	
Age					
>60 y	28 (25.9)	50 (46.3)	30 (27.8)	108	0.753
≤60 y	20 (30.3)	27 (40.9)	19 (28.8)	66	
Stage^2^					
I	14 (26.9)	31 (59.6)	7 (13.5)	52	0.008*
II	15 (22.7)	28 (42.4)	23 (34.8)	66	
III	18 (34.0)	16 (30.2)	19 (35.8)	53	
IV	1 (33.3)	2 (66.7)	0 (0.0)	3	
Tumor size					
≤5 cm	35 (26.5)	63 (47.7)	34 (25.8)	132	0.242
>5 cm	13 (31.0)	14 (33.3)	15 (35.7)	42	
Node metastasis					
Positive	27 (32.1)	27 (32.1)	30 (35.7)	84	0.007*
Negative	21 (23.3)	50 (55.6)	19 (21.1)	90	
Differentiation^3^					
Well	0 (0.0)	8 (100.0)	0 (0.0)	8	<0.001*
Moderate	28 (28.9)	49 (50.5)	20 (20.6)	97	
Poor	20 (33.9)	20 (33.9)	29 (32.2)	69	

1 The expression levels of DLK1 in tumor were evaluated by multiple pairwise comparisons with χ^2^ test between the groups below.

2 Stage I versus II + III + IV.

3 Well versus moderate versus high poor.

* *p* < 0.05 indicates statistical significant

**Table 3:** The expression of DLK1 versus clinical features in ADC

Clinicopathology	Immunohistochemical scoring of DLK1, *n* (%)			Total	*P*^1^
	Negative	Weak	Strong		
Gender					
Male	22 (24.7)	45 (50.6)	22 (24.7)	89	0.552
Female	18 (20.5)	52 (59.1)	18 (20.5)	88	
Age					
>60 y	20 (24.7)	45 (55.6)	16 (19.8)	81	0.657
≤60 y	20 (20.8)	52 (54.2)	24 (25.0)	96	
Stage^2^					
I	11 (16.4)	38 (56.7)	18 (26.9)	67	0.189
II	10 (23.8)	24 (57.1)	8 (19.0)	42	
III	18 (32.7)	24 (43.6)	13 (23.6)	55	
IV	1 (7.7)	11 (84.6)	1 (7.7)	13	
Tumor size					
≤5 cm	30 (20.5)	82 (56.2)	34 (23.3)	146	0.366
>5 cm	10 (32.3)	15 (48.4)	6 (19.4)	31	
Node metastasis					
Positive	18 (20.2)	49 (55.1)	22 (24.7)	89	0.520
Negative	22 (25.6)	48 (55.8)	16 (18.6)	86	
Missing				2	
Differentiation^3^					
Well	5 (20.8)	13 (54.2)	6 (25.0)	24	0.655
Moderate	19 (19.2)	56 (56.6)	10 (18.5)	99	
Poor	16 (29.6)	28 (51.9)	10 (18.5)	54	

1 The expression level of DLK1 in tumor were evaluated by multiple pairwise comparisons with χ^2^ test between the groups below.

2 Stage I versus II + III + IV.

3 Well versus moderate versus high poor.

### Abnormal nuclear translocation of DLK1 in tumor cells

**Figure 2:**
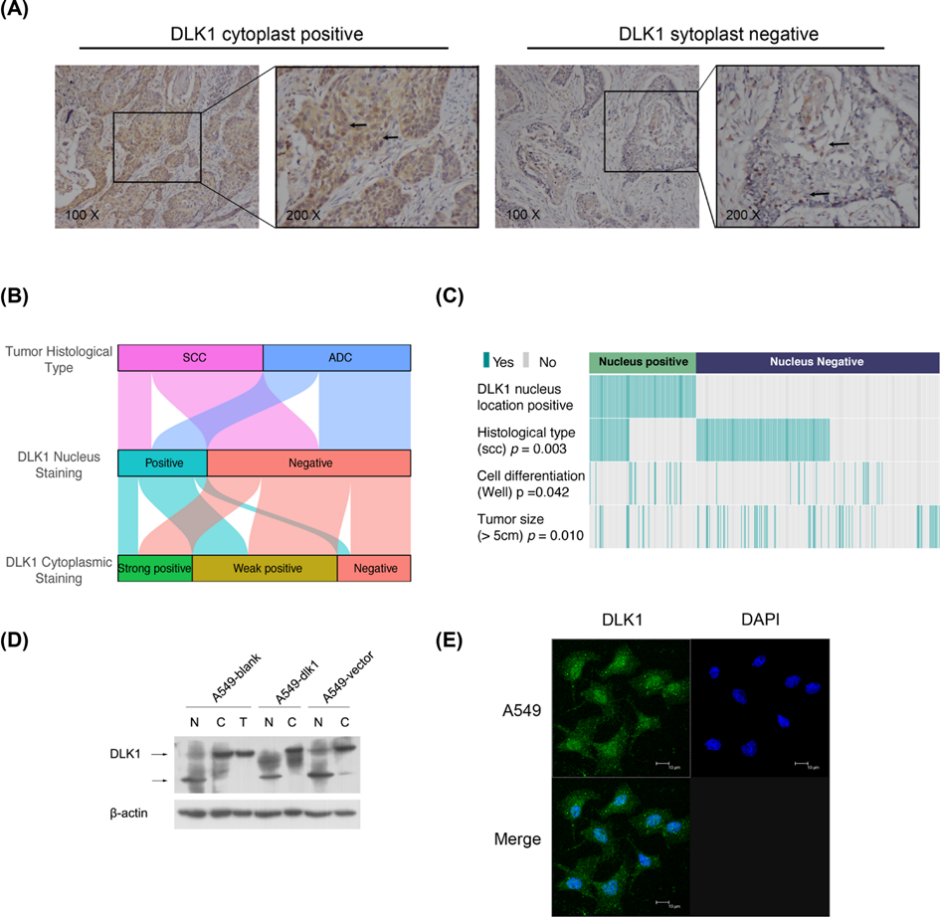
DLK1 is abnormally located in tumor cell nuclear (**A**) Representative staining of DLK1 in tumor cell nuclear in NSCLC tissues with cell cytoplast staining either positive or negative. (**B**) Graphical summary of DLK1 cytoplast staining, DLK1 nuclear staining and their histological type distribution. (**C**) Comparing different histologic types, cell differentiation and tumor size between DLK1 nuclear translocation positive or negative tumors. (**D**) Western blot of DLK1 in different cell components in A549 cell transfected with DLK1 plasmid (A549-dlk1), empty plasmid (A549-vector) and nothing (A549-blank) separately. N, nuclear proteins; C, cytoplasmic proteins; T, total protein. b-actin was used as the loading control. (**E**) Confocal microscopy analysis of endogenous DLK1 nuclear translocation in A549 cells. DLK1 was labeled with an Alexa Fluor® 488–conjugated second Ab and appears green; nuclear was labeled with DAPI and appears blue. In the merged image, colocation appears light blue.

Since DLK1 does not have an NLS domain, DLK1 was generally considered to be a cytoplasmic and membrane-binding protein and most studies commonly focus on its soluble or ligand isoform. Still, we could not ignore the factor that DLK1 staining was detected in 30.5% (107/351) NSCLC tumor cells (Figure 2A). Moreover, 16 cases (14.9%, 16/107) were found among DLK1 cytoplasmic staining negative samples. We summarized the distribution of DLK1 positive staining in either cell nuclear or cytoplasm, as well as the tumor histological type (Figure 2B). The proportion of DLK1 nuclear staining in ADC (37.9%, 67/177) was slightly higher than that in SCC (23.0%, 40/174). Coincidence of nuclear positive and cytoplasmic positive in ADC (33.3%, 59/177) was also higher than that in SCC (18.4%, 32/174). The proportion of samples with DLK1 nuclear positive staining among the DLK1 cytoplasmic positive staining is 34.6% (91/263), indicated that DLK1 nuclear expression might not be a synergistic event with cytoplasmic expression. We reanalyzed the IHC data, regardless of DLK1 cytoplasmic expression, only considering the association between DLK1 nuclear expression and the clinical features. Other than tumor histologic type, DLK1 nuclear expression was associated with cell differentiation (*P*=0.042) and tumor size (*P*=0.010). DLK1 nuclear expression was inclined to appear in well differentiated or smaller (less than 5 cm in diameter) tumor sample (Figure 2C).

To substantiate the observation, we examined the cellular localization of endogenous DLK1 in lung cancer cell lines. After isolating nuclear component from other cell organelle (cytoplasm, membrane etc.), proteins were extracted for Western blot detection of DLK1. As shown in Figure 2D, DLK1 had been detected in nuclear extracts in A549 cells, no matter transfected with DLK1 expression plasmid or not. The result indicated that DLK1 nuclear translocation was an endogenous phenomenon. Also, we could notice that the band size of nuclear localized DLK1 was smaller than that of cytoplasmic localized DLK1, which indicated that there should be modification or splicing for nuclear localized DLK1. Immunofluorescence was performed for intuitively characterizing nuclear translocation of DLK1 in tumor cells. In A549 cells, DLK1 was labeled by Alexa Fluor® 488–conjugated antibodies and appeared green. DLK1 was observed in nucleus. When merging with DAPI (blue), nuclear staining DLK1 turned into light blue.

### Identification of NCOR1 as DLK1 interacting protein in nucleus

**Figure 3:**
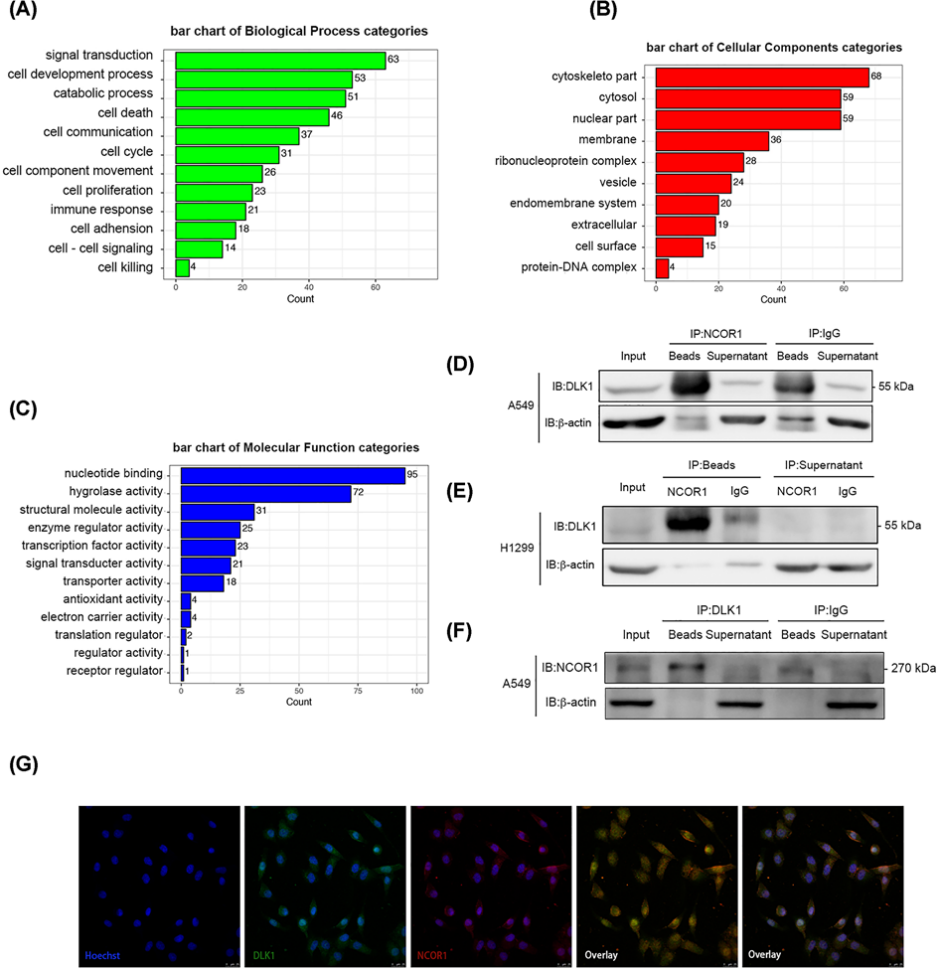
DLK1 interacts with NCOR1 in nucleus (**A–C**) GO annotation analysis for DLK1 pull-down proteome. y-axis shows the top enriched GO terms and x-axis shows the protein count in each GO term. (**D–F**) co-IP analysis of endogenous DLK1 with NCOR1. Input, nonimmunoprecipitation cell lysate; IgG, control IP with isotype antibody. IP indicated the antibodies used for IP; IB indicated the antibodies used for Western blotting. (**G**) Confocal microscopy analysis of DLK1 colocalization with NCOR1 in A549 cells. DLK1 was labeled with an Alexa Fluor® 488–conjugated second Ab and appears green; NCOR1 was labeled with Alexa Fluor®555–conjugated second Ab and appears red; nuclear was labeled with DAPI and appeared blue. In the merge image, colocation appear as orange (DLK1 + NCOR1) or purple (DLK1 + NCOR1 + DAPI).

To explore the mechanism of DLK1 nuclear translocation independent of NLS and discover its function in nucleus, we sought to identify DLK1 interacting proteins. Total protein extracts from A549 cells was incubated with DLK1 polyclonal rabbit antibody and pulled down with protein A/G beads. An isotype antibody of rabbit normal IgG was used as negative control. The associated proteins were analyzed by SDS/PAGE and Coomassie Brilliant Blue staining (Supplementary Figure S1). There were three specific bands in DLK1 pull-down samples. Together with their matched bands from negative control samples, which might not be visible, were excised and analyzed by mass spectrometry (MS). A total of 386 potential binding proteins were identified from two independent pull-down experiments. Gene ontology (GO) annotation (Figure 3A–C) and network analysis (Supplementary Figure S2) were performed to characterize the biological feature of potential binding proteins. It is worth noticing that 15.3% identified proteins were enriched in cell nucleus and 30.6% of them had nucleotide binding or transcription factor activity. Twelve proteins were detected in either replicates. We used co-immunoprecipitation (co-IP) and Western blot to validate their interaction with DLK1. Forward immunoprecipitation (IP) by DLK1 in A549 cells demonstrated that DLK1 could bind to NCOR1. Reverse IP by NCOR1 in A549 cells and H1299 cells demonstrated that NCOR1 could bind to DLK1 specifically (Figure 3D–F). To address whether DLK1 interacts with NCOR1 in nucleus, we performed dual immunofluorescent staining in A549 cells. DLK1 was labeled green and NCOR1 was labeled red. Their colocalization was merged in nucleus and appeared orange (Figure 3G).

### Suppression of NCOR1 in NSCLC indicated poor prognosis

NCOR1, a nuclear receptor, part of a complex which promotes histone deacetylation and the formation of repressive chromatin structures [18]. Participates in the transcriptional repressor activity, negatively regulates androgen receptor signaling and androgen-induced cell proliferation [19]. Several studies revealed that NCOR1 has the characteristics of tumor suppressor including glioblastoma, malignant melanoma and breast cancer [20–23].

**Figure 4:**
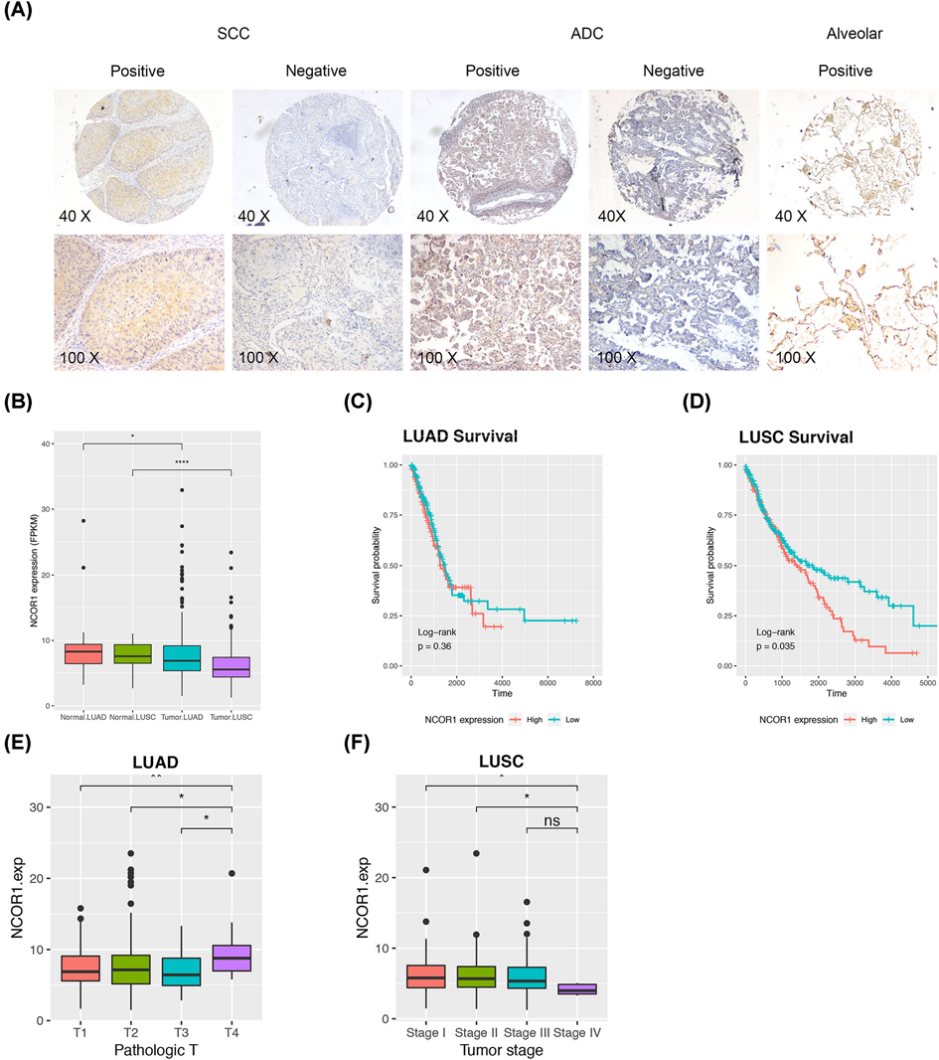
NCOR1 is down regulated in NSCLC (**A**) Representative staining of NCOR1 IHC in SCC and adenocarcinoma (ADC) tissue microarrays (TMA). (**B–F**) Datasets were downloaded from TCGA. (B) Boxplot for NCOR1 gene expression levels (FPKM) in NSCLC patients. Kaplan–Meier analysis for the overall survival of LUAD (C) and LUSC (D) patients with different expression levels of NCOR1. (E) NCOR1 gene expression was associated with pathologic T status in LUAD. (F) NCOR1 gene expression was associated with tumor stage in LUSC. Statistic method is wilcox.test; ns, not significant; *, *P*-value <0.05; **, *P*-value <0.01; ****, *P*-value <0.0001.

To determine the role of NCOR1 in NSCLC and explore the coordination with DLK1, we examined the expression of NCOR1 on the same batch of TMA samples by IHC. NCOR1 was significantly down-regulated in (55.1%) NSCLC specimens, while generally expressed in normal tissues (97.6%) (Figure 4A and Table 4). We investigated the correlation of NCOR1 expression and the clinical features of the samples, including patients’ age, gender, tumor histological type, tumor stage, tumor size, node metastasis status and cell differentiation degree. However, statistic significant difference was only found in tumor stage among ADC samples (Table 5). The expression of NCOR1 was dramatically reduced in advanced tumor samples (stages III and IV) compared with early stage tumor samples. Same trend existed in SCC, but with no statistical significance (data not shown).

**Table 4:** The expression of NCOR1 in NSCLC specimens

Histological type	Immunohistochemical scoring of DLK1, *n* (%)		Total	*P*
	Negative	Positive		
Tumor tissue	66 (44.9)	81 (55.1)	147	
Normal tissue	3 (2.4)	123 (97.6)	126	<0.001^1^

1 The expression of DLK1 in primary tumors versus normal lung tissues was significant, *P*<0.05 with χ^2^ test. The morphologically normal tissues were collected from the same group of lung cancer patients.

**Table 5:** The expression of NCOR1 versus clinical features in ADC

Clinicopathology	Immunohistochemical scoring of DLK1, *n* (%)		Total	*P*^1^
	Negative	Positive		
Gender				
Male	49 (45.8)	58 (54.2)	107	0.853
Female	17 (43.6)	22 (56.4)	39	
missing	1			
Age				
>60 y	46 (48.9)	48 (51.1)	94	0.228
≤60 y	20 (37.7)	33 (62.3)	53	
Stage^2^				
I	18 (34.0)	35 (66.0)	53	0.001*
II	13 (29.5)	31 (70.5)	44	
III	21 (61.8)	13 (38.2)	34	
IV	4 (66.7)	2 (33.3)	6	
Tumor size				
≤5 cm	51 (41.8)	71 (58.2)	122	0.123
>5 cm	15 (60.0)	10 (40.0)	25	
Node metastasis				
Positive	26 (45.6)	31 (54.4)	57	0.866
Negative	39 (43.8)	50 (56.2)	89	
Differentiation^3^				
Well	2 (25.0)	6 (75.0)	8	0.273
Moderate	55 (48.2)	59 (51.8)	114	
Poor	9 (36.0)	16 (64.0)	25	

1 The expression level of NCOR1 in tumor were evaluated by multiple pairwise comparisons with χ^2^ test between the groups below.

2 Stage I + II versus III + IV.

3 Well versus moderate versus high poor.

* *p* < 0.05 indicates statistical significant.

For more information, we downloaded RNA-seq data of NSCLC from TCGA to analyze the expression of NCOR1 in another dependent large scale cohort. LUSC and LUAD correspond to SCC and ADC in order to distinguish the sample source. The expression level of NCOR1 was reduced in tumor compared with normal, and the LUSC samples seemed to have smaller median value than the LUAD samples (Figure 4B). Survival analysis indicated NCOR1 could be a prognostic marker for LUSC patients (Figure 4C,D). Multivariate cox regression analysis revealed that NCOR1 was an independent prognosis factor for lung cancer (Supplementary Figure S3). We also investigated the correlation between NCOR1 expression and the clinical feature as was done on NCOR1 protein level (Supplementary Figure S4). In LUAD, the NCOR1 expression was raised accompanied with the advancement of pathologic T stage, which mainly stands for larger tumor size (Figure 4E). In LUSC, the negative correlation between NCOR1 expression and tumor stage was revealed. NCOR1 expression was down-regulated in stage IV samples (Figure 4F).

**Figure 5:**
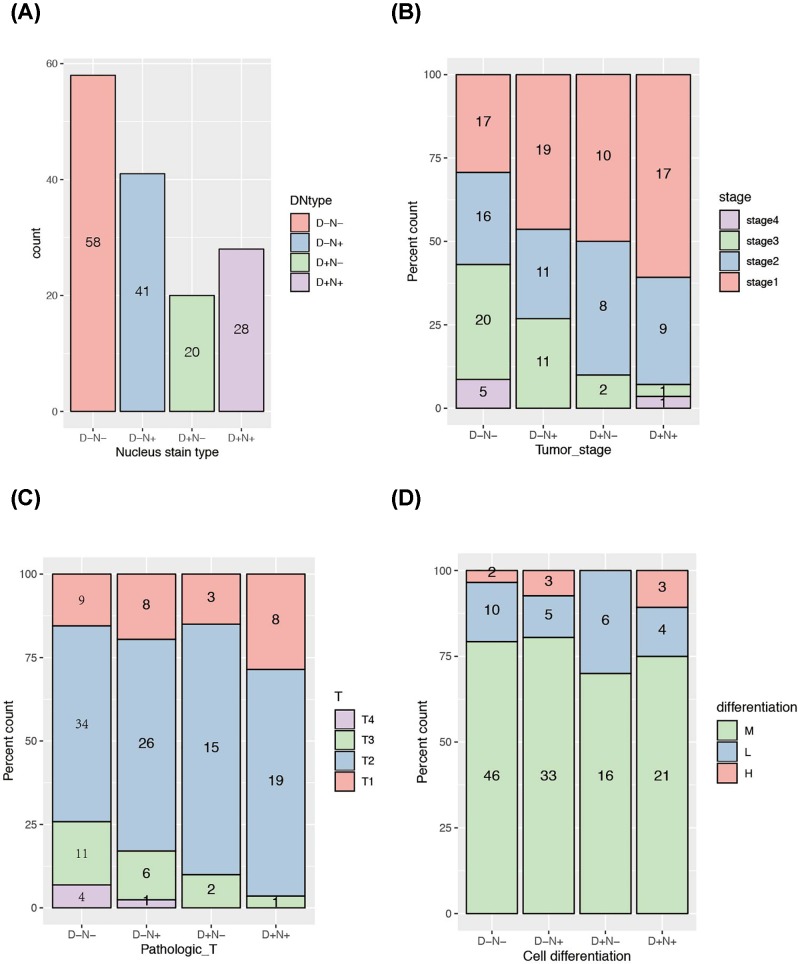
Comprehensive analysis of DLK1 and NCOR1 co-expression in tumor cell nuclear of NSCLC TMAs with clinical features Four conditions of DLK1 and NCOR1 nuclear staining is abbreviated as D+N−, for example, in which D stands for DLK1, N stands for NCOR1, ‘+’ stands for positive staining and ‘−’ stands for negative staining. The numbers inside the column indicates the sample number in each group. (**A**) The distribution of sample cases in different DN types. (**B–D**) Stack plot of sample cases of four DN types among tumor stage (B), pathologic T stage (C) and cell differentiation levels (D).

We evaluated that whether DLK1 and NCOR1 co-expression in nucleus is associated with any tumor character, the answer of which might give a hint on the mechanism of how DLK1 affects Notch signaling pathway. Four conditions of DLK1 and NCOR1 staining in nucleus were calculated (Figure 5) and we named them as four DN types, in which D represents DLK1 and N represents NCOR1. The distribution of four DN types is shown in Figure 5A. In total 147 tested samples, the D-N type samples are the most, with 58 cases, accounting for 39.5%; D+N− type samples are the least, with 20 cases, accounting for 13.6%. There were 28 cases of D+N+ type samples, which suggested that DLK1 and NCOR1 work together in nucleus, accounting for 19.0%.

We also analyzed the distribution of tumor stage, pathologic T stage and tissue differentiation in different DN types. From DN negative samples to DN positive samples, with the emergence of DLK1 and NCOR1 in the nucleus, the proportion of early tumor samples (stage I) significantly increased (*P*=0.013) (Figure 5B). In TNM staging system, T describes the size of the original tumor and whether it has invaded nearby tissue. We could observe that the T3/T4 samples are mainly concentrated in the D− negative samples, and with the emergence of DLK1 and NCOR1 in the nucleus, T3/T4 samples are getting less and less (Figure 5C).

The vast majority of the samples analyzed were moderately differentiated samples, resulting in a large bias in statistical analysis. Nonetheless, by comparing the ratio of high and low differentiated samples through the stack plot, we found that nuclear type of DLK1 and nuclear type of NCOR1 have a contradictory effect toward cell differentiation regulation. Setting D−N− type group as base line, D−N+ increased the ratio of high/low differentiated samples, while D−N+ reduced it. Such ratio was raised back in D+N+ group indicated that DLK1 and NCOR1 might have antagonistic effect in nucleus (Figure 5D).

## Discussion

DLK1 is a transmembrane protein famous for its interaction with NOTCH1. Although lack of DSL (Delta/Serrate/LAG-2) domain, known to conduct interactions between NOTCH receptors and canonical ligands, direct evidences showed that DLK1 could directly interact with NOTCH via DOS (Delta and OSM-11) domain or EGF domains [24]. Besides the impact of DLK1 on cell phenotype and tumorigenesis, the effect of DLK1 toward Notch signaling is also controversial. In summarized studies, the interaction of DLK1 and NOTCH1 either positively or negatively regulates Notch signaling [11,12,25–27].

Since the extracellular domain of DLK1 can be cleaved and released as a soluble protein, researchers have begun to pay attention to the functional difference between membrane- tethered isoform and the soluble isoform of DLK1. Garcés and co-workers [28] demonstrated that membrane-tethered DLK1 was essential for preadipocytic cells to receive and respond to differentiation signals. Likewise, in the brain study, DLK1 was shown to regulate stem cell numbers requires both membrane-tethered and soluble isoform [29]. Nueda and co-workers [27] hypothesized that the dual role of DLK1 was attributed to its isoforms. In this regard, the membrane-bound isoform may be particularly important since multiple studies demonstrated this isoform to exert the inhibiting effect on cell. This theory was supported in subsequent researches [13,30,31]. Until recently, Nueda and co-workers [9] discovered that high and low levels of DLK1 inversely affect breast cancer proliferation, adding to the complexity. When the existing assumptions do not fully support the existing observation, there must be details that we ignore. We observed that DLK1 has nuclear staining during IHC assay in NSCLC tissues. By reviewing the literature, we found that we are not the first the group who recorded the nuclear localization of DLK1, and this phenomenon did not happen accidentally in NCSLC tissues [16,17,24]. Unfortunately, researchers ignored it because DLK1 does not have NLS. This time, we chose to face this phenomenon, verify its authenticity and study the mechanisms behind it.

First of all, we calculated the positive rate of DLK1 nuclear staining among the immunohistochemical staining of 351 NSCLC tissues. The proportion of DLK1 nuclear staining was relatively high (30.5%). Moreover, this phenomenon has certain clinical significance involved in tissue differentiation and tumor size, consistent with the known isoforms. Some studies suggested that membrane-bound isoform and soluble isoform might play distinct roles in cell differentiation [30,32]. We can not help wondering with the situation when three isoforms are present. Therefore, we decided to validate the nuclear localization of DLK1. Through nuclear fraction extraction or *in situ* immunofluorescent labeling, we confirmed with the nuclear localization of DLK1 in lung cancer cell lines (Figure 2D,E). Meanwhile, Western blot result indicated that nuclear localized DLK1 is smaller than its full length at approximately 30 kDa. We speculated that the nucleus-localized DLK1 might be generated either from the cleavage of the intracellular part of DLK1, or from endocytosis of soluble isoform released by adjacent cells, or other process. Finding out the sequence of the nuclear isoform is the key to studying its origin and is our next research project.

Since the lack of NSL, DLK1 is not transported by a traditional process. We search for clues by identifying the working partner of DLK1. Previous studies have identified a number of DLK1 interacting proteins. However, among them are neither nuclear localization proteins nor transport-related proteins [33–35]. For this reason, we performed our own screening and identification experiment. Through co-IP combined MS screening, we identified NCOR1 as a novel interacting protein of DLK1. Their interaction in nuclear was then confirmed by confocal observation (Figure 3D–G).

NCOR1 associates with nuclear receptors and other transcription factors leading to transcriptional repression. The NCOR1 repressed proteins contain downstream effector proteins of Notch signaling. Studies have shown that interfering NCOR1 expression can enhance cell proliferation and invasion, which leads to tumor growth and metastasis [23,36]. There are few reports on NCOR1 in lung cancer study, therefore we performed an IHC assay in NSCLC tissue microarrays. The results showed that NCOR1 was abnormally down-regulated in tumor tissues than paired normal tissues, and was associated with tumor stage advance (Tables 4 and 5, Figure 4A). In addition, we used public data from TCGA to explore the RNA expression of NCOR1 in NSCLC patients. Consistent with our results, NCOR1 is down-regulated in tumor samples and has a prognosis potential in LUSC.

Since we performed the IHC of NCOR1 and DLK1 on the same batch of samples, we investigated the co-location of DLK1 and NCOR1 in tumor cells. The distribution of the four localization pattern in our samples revealed a certain trend, which was associated with tumor stage, tumor size and cell differentiation. In terms of tumor stage and size, DLK1 and NCOR1 showed a synergistic effect in nucleus. With the appearance of DLK1 and NCOR1 in the nucleus, the proportion of early tumor stage increased, and the proportion of large tumor size decreased. Especially, the effect of nuclear DLK1 and cytoplasmic DLK1 were opposite in promoting cell proliferation. In terms of cell differentiation, DLK1 and NCOR1 showed an antagonistic effect in nucleus. In a systematic review, researches speculate the actual role of DLK1 may be to function as a checkpoint to slow down proliferation while forcing cells into the process of differentiation [6]. According to our result, interaction of nuclear DLK1 and NCOR1 may also play an important role in such process. Due to the limitation of sample quality, there are bias during statistical analysis. This part of result should be supplemented and improved in the follow-up work by more accurate methods like dual-labeled immunofluorescence staining.

In conclusion, we verified the nuclear localization of DLK1 in lung cancer cells, for which there was a prior reason to expect. We also identified NCOR1 as DLK1’s working partner in nucleus and might participate in cell differentiation regulation.

## Supplementary Material

Supplementary Figures S1-S4

## Abbreviations

ADC/LUAD: lung adenocarcinoma
co-IP: co-immunoprecipitation
DLK1: Delta-like homolog 1
FPKM: Fragments per kilobase of transcript per million fragments mapped
HCC: hepatocellular carcinoma
HRP: horseradish peroxidase
IHC: immunohistochemistry
LUSC/SCC: lung squamous cell carcinoma
MS: mass spectrometry
NCOR1: nuclear receptor corepressor 1
NLS: nuclear localization signal
NSCLC: non-small cell lung cancer
TCGA: The Cancer Genome Atlas
TMA: tumor tissue microarray
TNM: TNM staging system
