# Supplementary material for: Abnormally localized DLK1 interacts with NCOR1 in non-small cell lung cancer cell nuclear
Source: Biosci Rep. 2019 Dec 13;39(12):BSR20192362. doi: 10.1042/BSR20192362 (PMC6911156; doi:10.1042/BSR20192362)
Supplement: Supplementary Figures S1-S4 [file BSR-2019-2362_supp.pdf]

Supplementary Figures

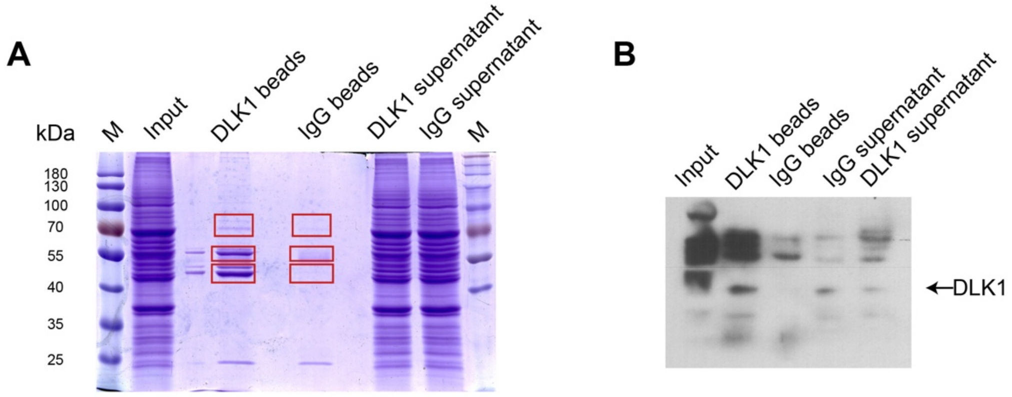

Figure. S1. DLK1 pull-down proteome preparation for mass spectrometry. (A) SDS-PAGE and Coomassie brilliant blue staining of proteins from DLK1 pull-down assay. Red box indicated the bands sent for mass spectrometry identification. (B) Western blot for the proteins from DLK1 pull-down assay. Arrow point out DLK1 band.

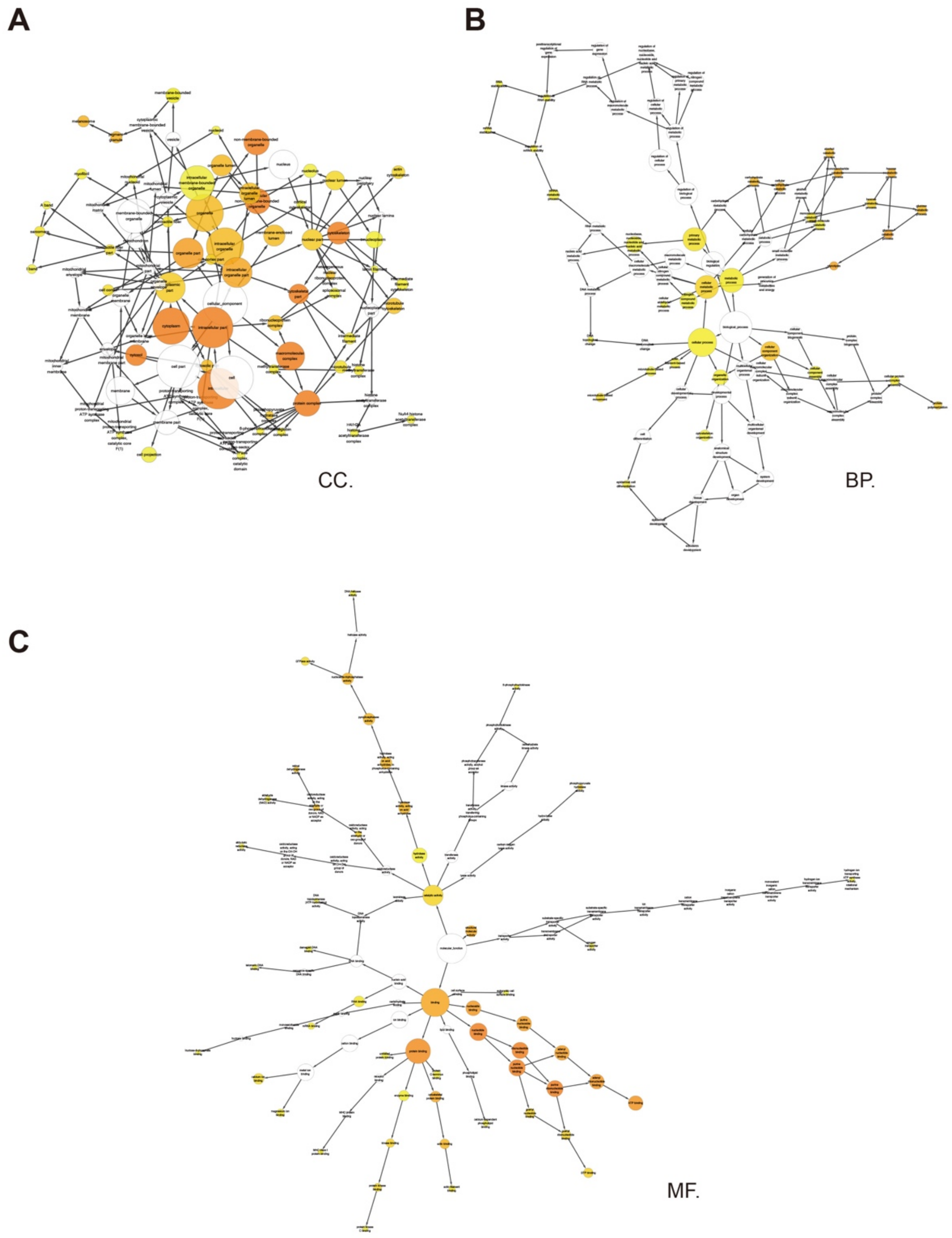

Figure. S2. Network analysis of the candidate proteins identified from mass spectrometry. Node size indicates the generality of GO term, node color indicates p value. CC, cell component; BP, biology process; MF, molecular function.

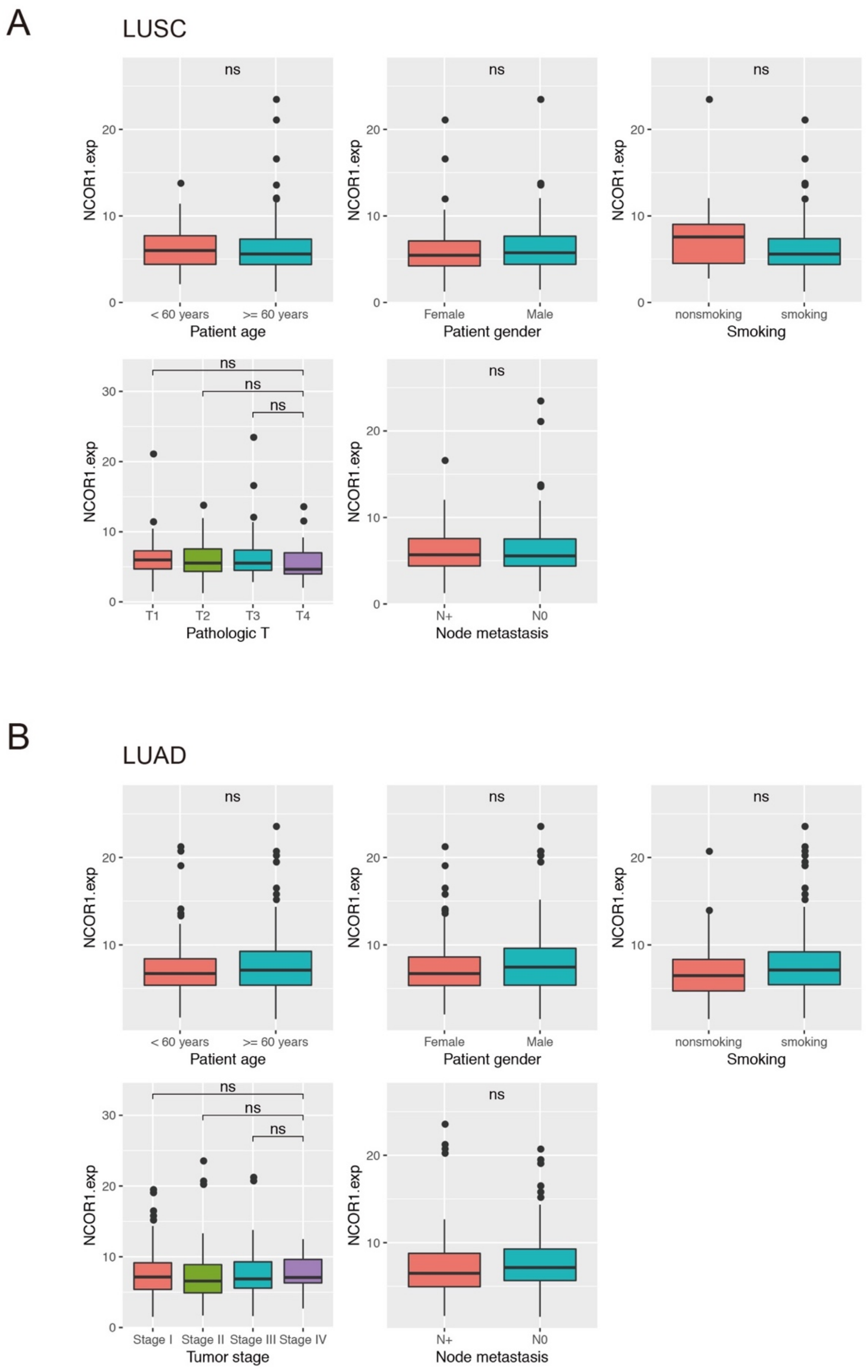

Figure. S3. Comprehensive analysis of NCOR1 expression and the clinical feature of the patients, including patient age, gender, smoking status, tumor stage, node metastasis status, pathologic T level in LUSC (A) and LUAD (B). Data sets were downloaded from the Cancer Genome Atlas(TCGA). Ns, not significant.

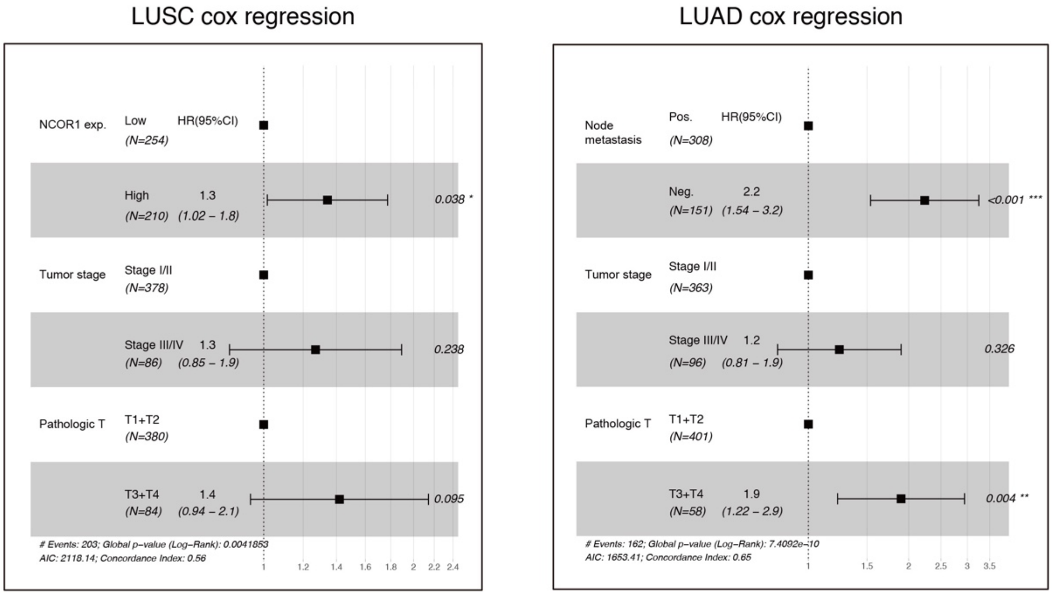

Figure. S4. Multivariate cox regression analysis for hazard of death (survival) in LUSC and LUAD. Data sets were downloaded from the Cancer Genome Atlas(TCGA). Patients with low NCOR1 expression showed reduced survival times compared with patients with high NCOR1 expression in LUSC(D).
